# Self-perception of aging and life satisfaction of Hispanic older adults: The role of family relationship

**DOI:** 10.1093/geroni/igaf122.3077

**Published:** 2025-12-31

**Authors:** Dahee Kim, Thuc Luong, Janet Lopez, Ayse Malatyali, Ladda Thiamwong, Abigail Tice, Yingru Li, Rui Xie

**Affiliations:** University of Central Florida, Orlando, Florida, United States; University of Central Florida, Orlando, Florida, United States; University of Central Florida, Orlando, Florida, United States; University of Central Florida, Orlando, Florida, United States; University of Central Florida, Orlando, Florida, United States; University of Central Florida, Orlando, Florida, United States; University of Central Florida, Orlando, Florida, United States; University of Central Florida, Orlando, Florida, United States

## Abstract

Individual self-perception of aging (SPA) is shaped by socio-cultural experiences across life stages and may affect emotional well-being in late adulthood. However, the association between SPA and emotional well-being in Hispanic older adults (HOA), as well as the contextual factors affecting this association, has not been explored. We aimed to (1) investigate the relationship between HOAs’ SPA and life satisfaction, one of emotional well-being indicators and (2) test the moderating effects of spousal relationships and relationship quality with children. We analyzed data of 143 HOAs from the Health and Retirement Study collected in 2016-2020. SPA was measured using the Attitudes Toward Own Aging (ATOA), and life satisfaction was measured using The Satisfaction with Life scale. Spousal relationship was assessed based on perceived closeness with spouses, and relationship quality with children was evaluated by the frequency of contact with children. The two moderation models were tested using SPSS PROCESS Macro. HOAs with more positive SPA were likely to have higher life satisfaction (r=.231, p<.01). In HOAs with less frequent contact with children, the more positive SPA, the higher life satisfaction they had. In contrast, in HOAs with more frequent contact with children, the more positive SPA, the lower life satisfaction they had (B=-.235, p<.05). There was no moderating effect of spousal relationships. This study highlights HOAs’ SPA and emotional well-being and showed frequent contact with children may have a negative effect on emotional well-being. Future research is expected to continue exploring the associations among HOA’s SPA, health/well-being indicators, and contextual factors.

